# Willingness to use a supervised injection facility among young adults who use prescription opioids non-medically: a cross-sectional study

**DOI:** 10.1186/s12954-017-0139-0

**Published:** 2017-02-20

**Authors:** Benjamin A. Bouvier, Beth Elston, Scott E. Hadland, Traci C. Green, Brandon D. L. Marshall

**Affiliations:** 1 Department of Epidemiology, Brown University School of Public Health, 121 South Main Street, Box G-S-121-2, Providence, RI 02912 USA; 2 Division of Adolescent/Young Adult Medicine, Department of Medicine, Boston Children’s Hospital, 333 Longwood Avenue, Boston, MA 02115 USA; 3 Department of Pediatrics, Harvard Medical School, 25 Shattuck Street, Boston, MA 02115 USA; 4 Department of Health Policy & Management, Harvard T. H. Chan School of Public Health, 677 Huntington Ave, Boston, MA 02115 USA; 5 Department of Emergency Medicine, Boston University School of Medicine, 771 Albany Street, Room 1208, Boston, MA 02118 USA; 6 The Warren Alpert School of Medicine of Brown University, Rhode Island Hospital, 55 Claverick Street, Providence, RI 02903 USA

**Keywords:** Supervised injection facility, Supervised injection site, Injection drug use, People who inject drugs, Prescription opioids, Opioid, Fentanyl, Harm reduction, Overdose, HIV/AIDS

## Abstract

**Background:**

Supervised injection facilities (SIFs) are legally sanctioned environments for people to inject drugs under medical supervision. SIFs currently operate in ten countries, but to date, no SIF has been opened in the USA. In light of increasing overdose mortality in the USA, this study evaluated willingness to use a SIF among youth who report non-medical prescription opioid (NMPO) use.

**Methods:**

Between January 2015 and February 2016, youth with recent NMPO use were recruited to participate in the Rhode Island Young Adult Prescription Drug Study (RAPiDS). We explored factors associated with willingness to use a SIF among participants who had injected drugs or were at risk of initiating injection drug use (defined as having a sex partner who injects drugs or having a close friend who injects).

**Results:**

Among 54 eligible participants, the median age was 26 (IQR = 24–28), 70.4% were male, and 74.1% were white. Among all participants, when asked if they would use a SIF, 63.0% answered “Yes”, 31.5% answered “No”, and 5.6% were unsure. Among the 31 participants reporting injection drug use in the last six months, 27 (87.1%) reported willingness to use a SIF; 15 of the 19 (78.9%) who injected less than daily reported willingness, while all 12 (100.0%) of the participants who injected daily reported willingness. Compared to participants who were unwilling or were unsure, participants willing to use a SIF were also more likely to have been homeless in the last six months, have accidentally overdosed, have used heroin, have used fentanyl non-medically, and typically use prescription opioids alone.

**Conclusions:**

Among young adults who use prescription opioids non-medically and inject drugs or are at risk of initiating injection drug use, more than six in ten reported willingness to use a SIF. Established risk factors for overdose, including homelessness, history of overdose, daily injection drug use, heroin use, and fentanyl misuse, were associated with higher SIF acceptability, indicating that young people at the highest risk of overdose might ultimately be the same individuals to use the facility. Supervised injection facilities merit consideration to reduce overdose mortality in the USA.

**Electronic supplementary material:**

The online version of this article (doi:10.1186/s12954-017-0139-0) contains supplementary material, which is available to authorized users.

## Background

Important public health and public safety concerns arise from injection drug use, including HIV transmission [1], hepatitis C virus (HCV) transmission [2, 3], overdose [3, 4], abscesses and infections [5, 6], and improperly discarded syringes [7]. Supervised injection facilities (SIFs)—also called drug consumption rooms (DCRs), safe injection sites (SISs), and medically supervised injection centers (MSICs)—have attempted to address these concerns by providing legally sanctioned, safer environments for people to inject pre-obtained drugs under medical supervision [8]. To date, more than 90 SIFs operate in ten countries (Australia, Canada, Denmark, France, Germany, Luxembourg, The Netherlands, Norway, Spain, and Switzerland) [9, 10]. Although there are different SIF models, facilities typically aim to provide clean injection equipment, education for safer injecting, medical response in the event of an overdose, and treatment referrals [8]. Many facilities offer a comprehensive array of health and social services, including detoxification and other substance use treatment services, medical care, counseling, and legal assistance [11, 12].

Over the last decade, the public health impacts of SIFs have been researched extensively. SIFs have been associated with reductions in overdose mortality [13], syringe sharing [14], unsafe injection practices [15], public injection drug use and public syringe disposal [16, 17], and demand for ambulance services [18]. SIF implementation has also been associated with increased referral to and uptake of detoxification and other substance use treatment services [19, 20]. Studies suggest that SIFs are cost-effective, potentially saving millions of dollars by preventing new HIV and HCV cases and overdose deaths [21–25], issues particularly relevant in the USA in recent years due to HIV outbreaks and the overdose crisis [26, 27]. A systematic review demonstrated that SIFs were not found to increase injection drug use, drug trafficking, or crime in surrounding environments [28]. Despite this body of scientific evidence, no SIFs have been implemented in the USA, primarily as a result of a complex set of national, state, and local policies and laws that forbid such facilities, most notably the so-called *Crack House Statute* of the federal *Controlled Substances Act*, which makes it unlawful to “knowingly open, lease, rent, use, or maintain any place, whether permanently or temporarily, for the purpose of manufacturing, distributing, or using any controlled substance” [29].

The state of Rhode Island, a small state in the Northeast United States, has experienced a dramatic increase in overdose deaths in the last decade, and now has one of the highest per capita overdose mortality rates in the country [30]. Particularly concerning is a surge in overdose deaths in the last 3 years due to illicitly manufactured fentanyl, a synthetic opioid that is 50–100 times more potent than morphine [31, 32]. Accidental overdose and death can be more likely to occur if individuals knowingly use fentanyl or unknowingly use other substances that have been adulterated with fentanyl [33]. A large proportion (47%) of overdose deaths in Rhode Island was attributable to fentanyl in 2015, and preliminary data from March to May of 2016 suggests that this proportion has continued to increase [34]. In Rhode Island, a wide array of stakeholders has collaborated to facilitate community responses to the overdose and fentanyl epidemic and to increase access to naloxone and to treatment and recovery services, but more efforts are needed to prevent overdose morbidity and mortality [35]. In general, while important successes have been achieved in the drug policy reform and harm reduction movements, drug policy in the USA has until very recently been framed as having zero tolerance/abstinence as the goal, with addiction largely treated as a criminal justice issue instead of as a public health one [36]. Despite the mounting evidence that harm reduction interventions such as syringe services programs are highly effective at preventing an array of drug-related harms, the implementation of many evidence-based programs, including SIFs, is non-existent or lacking in the USA [36].

Researchers and community organizations often conduct assessments of the prevalence and correlates of willingness to use a SIF to help provide a case for SIF implementation. Various studies have reported a high prevalence of willingness to use a SIF among people who inject drugs (PWID), and a number of factors related to willingness have been identified, including injecting in public, daily injection drug use, frequency of heroin use, ever experiencing an overdose, and familiarity with the concept of a SIF [37–47]. Studies have also shown that, once implemented, SIFs do indeed attract people at high risk of overdose, including those who inject in public and others at risk of blood-borne infection transmission [48–50]. However, to the best of our knowledge, only one peer-reviewed study has explored willingness to use a SIF among a sample in the USA. A study published in 2010 recruited PWID in San Francisco, California, and reported that 85% of participants reported willingness to use a SIF; having injected in public and having injected speedballs were associated with willingness [41]. In addition, to our best knowledge, no study has explored willingness to use a SIF in the context of the recent surge in fentanyl-attributable overdose deaths in North America [32]. Willingness to use a SIF in Rhode Island should be studied given its high per capita overdose mortality rate, its high proportion of fentanyl-attributable overdose deaths, and recent mobilization to respond to the overdose and opioid epidemics in the state [30, 31, 33–35].

This analysis uses data from the Rhode Island Young Adult Prescription Drug Study (RAPiDS), a pilot study that recruited youth aged 18 to 29 in Rhode Island who reported non-medical prescription opioid (NMPO) use. Between 2000 and 2014, the rates of death from prescription opioid overdose in the USA nearly quadrupled [51]. The RAPiDS study explored NMPO use among young adults in Rhode Island to help inform overdose prevention and other harm reduction interventions for this vulnerable population. We aim to report the prevalence of willingness to use a SIF among young adults who use prescription opioids non-medically. We also explore participant characteristics associated with higher willingness to use a SIF.

## Methods

### Participants

Young-adult NMPO users were invited to participate in RAPiDS between January 2015 and February 2016. Participants were recruited through targeted canvassing (e.g., bus advertisement, flyer), internet-based recruitment (e.g., posting to online classifieds such as craigslist), and word of mouth. Eligibility criteria were (1) currently living in Rhode Island, (2) between 18 and 29 years of age, (3) able to provide informed consent, (4) able to speak and feel comfortable completing a survey in English, and (5) report NMPO use in the past 30 days. Research staff screened interested individuals, and if eligible, participants completed an interviewer-administered survey in a public location of their choosing or at the Brown University School of Public Health. The survey lasted about 45 min, after which participants were compensated $25 USD. RAPiDS was approved by the Brown University Institutional Review Board (Protocol Entitled: RAPiDS: The Rhode Island Young Adult Prescription Study (#1403001006)).

Questions about SIF willingness and acceptability were added midway through the study period; thus, only 98 of the 200 enrolled participants were asked these questions. We sought to assess willingness to use a SIF among the participants who inject drugs or are at risk of initiating injection drug use, which we defined as having a sex partner or close friend who injects drugs. We decided to include participants who had not injected drugs but who had a sex partner or close friend who injects drugs because previous research has shown these persons to have a high risk of initiating injection drug use [52]. Moreover, exploring willingness to use a SIF among people at risk of initiating injecting might be useful in terms of gaging willingness to use a SIF among potential future injectors, and due to the fact that some SIFs (i.e., supervised consumption facilities) permit non-injection drug use [11, 53]. Thus, to be included in this analysis, participants had to be asked questions about SIF willingness and meet one or more of the following criteria: (1) have ever injected drugs, (2) have a sex partner who injects drugs, and (3) have at least one close friend who injects drugs. Among the 98 participants who were asked questions about SIF willingness, 34 participants had ever injected drugs, and 20 of the participants who had never injected drugs reported they had a sex partner who injects drugs and/or had at least one close friend who injects drugs. These 54 participants constitute the sample of this analysis. The other 44 participants had never injected drugs nor did they report having a sex partner or close friend who injects drugs, so they were excluded from the analytic sample. Thus, the total number of excluded participants was thus 146: 102 were not asked questions about SIF willingness, and 44 were asked questions about SIF willingness but had not injected drugs and did not have a sex partner or close friend who injects. In a supplementary analysis, we compared the demographics of eligible participants with excluded participants (see Additional file 1).

### Measures and statistical analyses

First, we ascertained whether participants would use a SIF service if one were available. Participants were asked, “If there was a legal service you could go to for free to inject safely indoors, would you use this service?” Possible responses were, “yes”, “no”, and “don’t know/refuse”. Then, we examined the acceptability of a SIF service among the participants who reported they would use the service. These questions included how often one would use a SIF (once or a couple times, about once a month, at least every week, every day), the longest time willing to travel to a SIF (1–5, 6–10, 11–20, 21–30 min, more than 30 min), and time of the day most likely to use a SIF (8 a.m.–12 p.m., 12 p.m.–4 p.m., 4 p.m.–8 p.m., 8 p.m.–12 midnight). We also asked participants who had ever injected drugs why they or their friends have needed help injecting (new user, don’t know how, bad veins/no veins, hate needles/afraid, too high/dopesick, shaky hands, other), since staff at SIFs often educate attendees on the best injection practices, so reasons for needing help to inject might be relevant when reporting on willingness to use a SIF. These questions were guided by previous research exploring the acceptability of SIF services. We report the number and proportion of participants who endorsed each answer choice. Finally, we explored factors associated with willingness to use a SIF, comparing participants who reported they were willing to use a SIF vs. participants who were unwilling or unsure using Fisher’s exact test and the Wilcoxon rank sum test. The factors we explored were age, sex at birth (male vs. female), race (categorized as white, black, or mixed/other), Hispanic or Latino descent, experiencing homelessness in the last six months (defined as not having a regular place to stay, living in a shelter because of nowhere else to go, or living in a place not ordinarily used for sleeping), ever overdosing by accident, frequency of injection drug use (≥daily vs. <daily), ever borrowing a used syringe/needle and ever lending a used syringe/needle, respectively (among the participants who reported a history of injection drug use), typically using prescription opioids non-medically alone, and ever using heroin. We also ascertained whether participants had used diverted fentanyl non-medically in the preceding six months (i.e., participants were asked if they had used fentanyl in the form of skin patches, pills, nasal sprays, intravenous (IV) fentanyl, lozenges/lollipops, and/or films/tablets without doctor’s orders or not as a doctor directed in the preceding six months). Finally, we also asked participants how often they used molly/MDMA/ecstasy, mushrooms, GHB, ketamine, crystal methamphetamine, and cocaine in the last six months (never, once or a couple times, about once per month, at least every week, every day); participants were considered to have engaged in opioid and non-opioid illicit poly-substance use at least once per month in the last six months if they reported that they used at least one of these substances at least once per month. These variables were guided by previous research on willingness to use a SIF and by previous research on risk factors for overdose. All analyses were conducted in SAS version 9.3, and all *p* values were two-sided. *P* values less than 0.05 were considered significant.

## Results

### Characteristics of participants

Among the 54 eligible participants, the median age was 26 (IQR = 24–28) and 38 (70.4%) were male. The majority (74.1%) was white, 7.4% were black, and 14.8% were mixed, bi-racial, multi-racial, or reported “Other”. Six participants (11.1%) reported Hispanic or Latino descent. Over one quarter (27.8%, *n* = 15) had been homeless in the preceding six months. Among the 34 participants who had ever injected drugs, 52.9% (*n* = 18) had ever borrowed a used syringe/needle and 52.9% (*n* = 18) had ever lent a used syringe/needle. Other sociodemographic characteristics of the study sample are shown in Table 1. A comparison of the characteristics of the eligible sample vs. those who were excluded is shown in the Additional file 1. Eligible participants were more likely to be younger (median age 26 vs. 24, *p* = 0.003), be white (74.1 vs. 56.8%, *p* = 0.034), and have accidentally overdosed (38.9 vs. 21.9%, *p* = 0.019).

**Table 1:** Factors associated with willingness to use a SIF among young adult non-medical prescription opioid users in Rhode Island

	Total	Yes	No or unsure	*p* value
	*n* (%)	*n* (%)	*n* (%)	
	*n* = 54	*n* = 34	*n* = 20	
Age (median, IQR)	26 (24–28)	26.5 (24–28)	25 (22.5–28)	0.31
Sex at birth				
Male	38 (70.4)	24 (70.6)	14 (70.0)	1.00
Female	16 (29.6)	10 (29.4)	6 (30.0)	
Race				
Black, African, Haitian, or Cape Verdean	4 (7.4)	1 (2.9)	3 (15.0)	0.16
White	40 (74.1)	27 (79.4)	13 (65.0)	
Aggregated “Mixed/Other”	8 (14.8)	4 (11.8)	4 (20.0)	
Ethnicity				
Not Hispanic or Latino	48 (88.9)	29 (85.3)	19 (95.0)	0.39
Hispanic or Latino	6 (11.1)	5 (14.7)	1 (5.0)	
Been homeless in the last six months				
Yes	15 (27.8)	14 (41.2)	1 (5.0)	<0.01
No	39 (72.2)	20 (58.8)	19 (95.0)	
Ever overdosed by accident				
Yes	21 (38.9)	17 (50.0)	4 (20.0)	0.04
No	33 (61.1)	17 (50.0)	16 (80.0)	
Daily injection drug use				
Yes	12 (22.2)	12 (35.3)	0 (0.0)	<0.01
No^a^	42 (77.8)	22 (64.7)	20 (100.0)	
Ever borrowed a used syringe/needle^b^				
Yes	18 (52.9)	16 (57.1)	2 (40.0)	0.64
No	15 (44.1)	12 (42.9)	3 (60.0)	
Ever lent a used syringe/needle^b^				
Yes	18 (52.9)	17 (60.7)	1 (20.0)	0.15
No	15 (44.1)	11 (39.3)	4 (80.0)	
Typically use prescription opioids non-medically alone				
Yes	44 (81.5)	31 (91.2)	13 (65.0)	0.03
No	10 (18.5)	3 (8.8)	7 (35.0)	
Ever used heroin				
Yes	36 (66.7)	29 (85.3)	7 (35.0)	<0.01
No	18 (33.3)	5 (14.7)	13 (65.0)	
Ever used diverted fentanyl non-medically				
Yes	20 (37.0)	17 (50.0)	3 (15.0)	0.02
No	34 (63.0)	17 (50.0)	17 (85.0)	
Illicit poly-substance use at least once per month in the last six months				
Yes	28 (51.9)	20 (58.8)	8 (40.0)	0.26
No	26 (48.1)	14 (41.2)	12 (60.0)	

^a^Category includes 19 participants who injected in the last six months but less than daily, 3 participants who reported lifetime injection drug use but none in the last six months, and 20 participants who reported no lifetime injection drug use (but were considered to be at high risk of initiating injection drug use)

^b^Among the 34 participants who had ever injected drugs

^c^ Not all columns add to 100% due to missing values

Risk factors for overdose were prevalent: for example, a majority (81.5%) reported using prescription opioids non-medically alone, 66.7% had ever used heroin, and 37.0% had ever used diverted fentanyl non-medically. Twenty-one (38.9%) participants had ever accidentally overdosed, 11 (52.4%) of whom had accidentally overdosed in the last six months.

### Prevalence and correlates of willingness to use a supervised injection facility

When asked if they would use a SIF service, 63.0% (*n* = 34) answered “Yes”, 31.5% (*n* = 17) answered “No”, and 5.6% (*n* = 3) were unsure. A majority (74.1%, *n* = 40) thought their friends or other people they knew would use a SIF. Among the participants who reported willingness to use a SIF (*n* = 34), the most commonly endorsed answer for how often he/she would use a SIF was “every day” (38.2%, *n* = 13), and the second most commonly endorsed answer was “at least every week” (26.5%, *n* = 9). The most commonly endorsed answer for time most likely to use the service was 8 a.m.–12 p.m. (61.8%, *n* = 21), and the second most commonly endorsed answer was 12 p.m.–4 p.m. (35.3%, *n* = 12). The most commonly endorsed answer for why participants or participants’ friends have needed help injecting was “Bad veins/no veins.” Among the participants who were willing to use a SIF, 31 (91.2%) stated that their friends would also be willing to use this service. The responses to other acceptability questions are shown in Table 2.

**Table 2:** Acceptability of a SIF service among participants who reported willingness to use a SIF (*n* = 34)

	*n* (%)
How often would you use a SIF?	
Once or a couple of times	8 (23.5)
About once a month	3 (8.8)
At least every week	9 (26.5)
Every day	13 (38.2)
Don’t know	1 (2.9)
Longest time willing to travel to a SIF	
1–10 min	6 (17.7)
11–20 min	12 (35.3)
21–30 min	8 (23.5)
More than 30 min	6 (17.7)
Don’t know	2 (5.9)
When would you be most likely to use a SIF?^a^	
Mornings, like 8 a.m.–12 p.m.	21 (61.8)
Afternoons, like 12 p.m.–4 p.m.	12 (35.3)
Evenings, like 4 p.m.–8 p.m.	10 (29.4)
Late evenings, like 8 p.m.–12 midnight	7 (20.6)
Why have you or your friends needed help injecting?^b^	
New user	13 (46.4)
Don’t know how	15 (53.6)
Bad veins/no veins	18 (64.3)
Hate needles/afraid	6 (21.4)
Too high/dopesick	9 (32.1)
Shaky hands	7 (25.0)
Other	3 (10.7)
Do you think that your friends (or other people you know) would use this service?	
Yes	31 (91.2)
No	2 (5.9)
Don’t know	1 (2.9)

^a^Column adds to >34 because this question was “check all that apply”

^b^Among the 28 participants who had ever injected drugs. Column adds to >28 because this question was “check all that apply”

Willingness to use a SIF increased with frequency of injection drug use: two of the three (66.7%) participants who had ever injected but not in the last six months reported willingness, and 15 of the 19 (78.9%) participants who injected less than daily in the last six months reported willingness, while all 12 (100.0%) of the participants who injected daily or multiple times a day in the last six months reported willingness. Overall, 27 (87.1%) of the 31 participants who had injected drugs in the last six months reported willingness. A total of five (25.0%) of the 20 participants who had never injected drugs (but who had a sex partner or close friend who injects) reported willingness to use a SIF.

As shown in Table 1, compared to participants who were unwilling or were unsure, participants who reported willingness to use a SIF were more likely to have been homeless in the preceding six months (41.2 vs. 5.0%, *p* = 0.004), have ever accidentally overdosed (50.0 vs. 20.0%, *p* = 0.043), report daily injection drug use (35.3 vs. 0.0%, *p* < 0.001), have ever used heroin (85.3 vs. 35.0%, *p* < 0.001), have ever used diverted fentanyl non-medically (50.0 vs. 15.0%, *p* = 0.018), and typically use prescription opioids alone (91.2 vs. 65.0%, *p* = 0.028).

## Discussion

Our study confirms the findings of other studies that have reported a high prevalence of willingness to use a SIF among people who inject drugs [37–42, 45, 46]. Because we included participants who had never injected drugs but were at high risk of initiating injection drug use in our analysis, we note that the overall willingness may be lower than that reported by other studies. It is also important to note that our sample is different from other studies that have explored willingness to use a SIF: whereas previous studies have recruited people who inject drugs, the sample for this analysis is comprised of people who reported past 30-day NMPO use and who inject drugs or have a sex partner or close friend who injects drugs.

Our analyses support previous research on the correlates of SIF acceptability by confirming that variables such as having used heroin and having accidentally overdosed are associated with higher acceptability [37–40, 45, 46]. We also observed correlates of willingness we believe have not yet been identified. Notably, of those willing, 50% reported ever using diverted fentanyl non-medically. This finding demonstrates that SIFs may be an effective approach to reduce the high rate of fentanyl-attributable fatal overdoses observed in Rhode Island and in other regions throughout the USA [54, 55]. For decades, United States drug policy has focused on combating drug trafficking, yet illicit drug prices have decreased, rates of drug use (specifically heroin use) are increasing, and overdose mortality rates are at an all-time high [26, 36]. Further exacerbating overdose risk is the increasing presence of potent contaminants (notably fentanyl) in heroin, counterfeit pills, and other drugs [32, 56, 57]. In this context, it is very unlikely that continuing to pursue “supply side” interventions and zero tolerance/abstinence-based policies will address this public health issue in an effective way [36, 58]. Rather, robust harm reduction programming and increased access to evidence-based treatment services are more paramount than ever to address the rising incidence of fentanyl-attributable overdose deaths [32, 59].

It has been proposed that there are various strategies involving local and state law to launch a SIF, but such a facility could only proceed free of legal uncertainty with federal authorities’ explicit authorization or decision to not interfere [60–62]. Nonetheless, efforts to open a SIF have been launched in multiple US cities, including San Francisco, Seattle, New York City, Baltimore, and Ithaca, New York [63–66]. A nonprofit organization in Boston recently established a supervised environment in which people can be medically monitored after using street drugs but cannot use drugs on the premises [67]. The New York City Council recently allocated $100,000 to the New York City health department to study the feasibility and impact of a SIF in the city [68]. Our results further demonstrate that efforts to establish a SIF in the USA are supported by a growing body of scientific evidence, and that persons at high risk of overdose would likely be willing to use such a facility.

Our study has important limitations that should be considered when interpreting our findings. First, our pilot study had a small sample size (*n* = 54) and thus limited statistical power. Because of this small sample size, we were not able to conduct any multivariate analyses; thus, observed associations may be confounded. Larger studies are needed to identify the independent characteristics associated with higher willingness to use a SIF. Second, only 54 of the 200 RAPiDS participants were asked questions regarding willingness to use a SIF and met our eligibility criteria. Eligible participants were more likely to be younger, be white, have ever been homeless, have been incarcerated, and have accidentally overdosed. It is possible that our sample is not representative of the full study in other important ways. Although we used multiple strategies to recruit participants, it is possible our findings may not be generalizable to all young adults who use prescription opioids non-medically in Rhode Island. Third, the age range of our participants (18–29) is small, especially considering the aging of people who inject drugs in the USA [69]. In addition, the primary goal of our study was to recruit people who reported NMPO use, whereas other populations of people who inject drugs might use a SIF. These eligibility criteria thus reduce the generalizability of our study to all people in Rhode Island who might use a SIF. Fourth, our survey asked if participants would use a hypothetical facility where people could inject drugs but did not ascertain whether participants had heard about SIFs before the survey. Additionally, participants were not asked why they were willing or not willing to use such a facility or to explain their responses to questions about preferences of a hypothetical SIF service. For example, our finding that mornings (8 a.m.–12 p.m.) and afternoons (12 p.m.–4 p.m.) were the most commonly endorsed answer choices for times most likely to use a SIF contradicts previous qualitative research that has reported that people use SIFs more often later in the day [70]. It is possible that some participants only use opioids once daily in the morning to get through the day and/or to avoid withdrawal symptoms. More research is required to further explore willingness to use a SIF and participant preferences of a potential SIF service. Furthermore, our survey asked specifically about a facility where people could inject drugs; additional studies are needed to further explore willingness to use a supervised consumption facility for non-injection drug use among individuals who do not inject drugs but are at high risk of overdose, including those who use diverted fentanyl non-medically [11, 53]. Finally, we relied on self-reported willingness to use a hypothetical SIF service. While it has been found that reported willingness measures collected before SIF opening independently predicted later attendance [71], it is important to note the potential invalidity of reporting willingness to use a hypothetical SIF.

## Conclusions

In summary, this study represents an early step in exploring the feasibility of implementing a SIF in the state of Rhode Island. If a SIF were opened, more than six in ten of our study participants reported they would use the service, and more than eight in ten of participants who have injected drugs reported they would use the service. Established risk factors for overdose were associated with higher SIF acceptability, indicating that young people at the highest risk of overdose might ultimately be the same individuals to use the facility. We also identified novel factors associated with higher willingness to use a SIF, including use of diverted fentanyl non-medically. Among the participants who reported willingness to use a SIF, the majority stated they would use the facility at least once a week. These data indicate that SIFs merit consideration to reduce overdose mortality among young adults who use prescription opioids non-medically and who inject drugs or are at risk of initiating injection drug use in the USA.

## Additional file

Additional file 1: Comparing the 54 participants eligible for this analysis and the 146 excluded participants. (DOCX 17 kb)

## Abbreviations

HCV: Hepatitis C virus
NMPO: Non-medical prescription opioid
PWID: People who inject drugs
RAPiDS: Rhode Island Young Adult Prescription Drug Study
SIF: Supervised injection facility
