# Supplementary material for: Willingness to use a supervised injection facility among young adults who use prescription opioids non-medically: a cross-sectional study
Source: Harm Reduct J. 2017 Feb 20;14:13. doi: 10.1186/s12954-017-0139-0 (PMC5319157; doi:10.1186/s12954-017-0139-0)
Supplement: Additional file 1: — Comparing the 54 participants eligible for this analysis and the 146 excluded participants. (DOCX 17 kb) [file 12954_2017_139_MOESM1_ESM.docx]

| **Additional file 1:** Comparing the 54 participants eligible for this analysis and the 146 excluded participants**^†^** | | | |
| --- | --- | --- | --- |
|  | **Eligible**  **(n=54)**  **n (%)** | **Excluded**  **(n=146)**  **n (%)** | ***p* - value** |
| **Age (median, IQR)** | 26  IQR=24-28 | 24  IQR=21-27 | <0.01 |
| **Sex at birth** |  |  |  |
| Male | 38 (70.4) | 93 (63.7) | 0.41 |
| Female | 16 (29.6) | 53 (36.3) |  |
| **Race** |  |  |  |
| Black, African, Haitian, or Cape Verdean | 4 (7.4) | 29 (19.9) | 0.03 |
| White | 40 (74.1) | 83 (56.8) |  |
| Aggregated “Mixed/Other” | 8 (14.8) | 33 (22.6) |  |
| **Ethnicity** |  |  |  |
| Not Hispanic or Latino | 48 (88.9) | 124 (84.9) | 0.65 |
| Hispanic or Latino | 6 (11.1) | 22 (15.1) |  |
| **Been homeless in the last 6 months** |  |  |  |
| Yes | 15 (27.8) | 35 (24.0) | 0.59 |
| No | 39 (72.2) | 111 (76.0) |  |
| **Ever overdosed by accident** |  |  |  |
| Yes | 21 (38.9) | 32 (21.9) | 0.02 |
| No | 33 (61.1) | 114 (78.1) |  |
| § Not all columns add to 100% due to missing values  **†** The total number of excluded participants was 146: 102 were not asked questions about SIF willingness, and 44 were asked questions about SIF willingness but had not injected drugs and did not have a sex partner or close friend who injects | | | |
